# Development of the high angular resolution 360° LiDAR based on scanning MEMS mirror

**DOI:** 10.1038/s41598-022-26394-6

**Published:** 2023-01-27

**Authors:** Donghai Yang, Yifan Liu, Qingjiu Chen, Meng Chen, Shaodong Zhan, Nim-kwan Cheung, Ho-Yin Chan, Zhidong Wang, Wen Jung Li

**Affiliations:** 1 Department of Mechanical Engineering, The City University of Hong Kong, Kowloon Tong, Hong Kong China; 2 AIphotonics Limited, Hong Kong Science and Technology Park, Shatin, Hong Kong China; 3 Faculty of Advanced Engineering, Chiba Institute of Technology, Chiba, Japan; 4 Present Address: GenHigh Tech Co., Limited, Nan Shan District, Shen Zhen, China

**Keywords:** Engineering, Mechanical engineering

## Abstract

Light detection and ranging (LiDAR) using various operational principles has been applied in many fields, e.g., robotics navigation, autonomous vehicles, unmanned aerial flyers, land surveying, etc. The multichannel LiDAR system is of great importance in the field of autonomous driving due to its larger field of view (FoV). However, the number of transceivers limits the vertical angular resolution of multichannel LiDAR systems and makes them costly. On the other hand, the emergence of microelectromechanical systems (MEMS) mirrors may provide a highly promising solution to a low-cost, high angular resolution LiDAR system. We have demonstrated a MEMS mirror-based 360° LiDAR system with high angular resolution and will present the detailed design process and obtained experimental results in this paper. With the combination of the MEMS mirror and a rotation platform for the LiDAR system, a 360° × 8.6° (horizontal × vertical) FoV was achieved. Compared with existing commercial multichannel 360° LiDAR systems, our system has 13.8 times better angular resolution than the Velodyne HDL-64 LiDAR sensor. The experimental results verified an excellent performance of 0.07° × 0.027° (horizontal × vertical) angular resolution, which enhances the panoramic scanning and imaging capability of the LiDAR system, potentially providing more accurate 3D scanning applications in areas such as autonomous vehicles, indoor surveying, indoor robotics navigation, etc.

## Introduction

LiDAR has been a well-known sensing technique in the past few decades and typically operates with a simple principle based on counting the elapsed time from the transmitted source pulse to the reflected received pulse, with a data processing unit calculating the distance between the pulse source and the object that reflected the pulse. The principle of LiDAR was first used for lunar ranging in 1962, and since then, many LiDAR systems have been developed for various applications, including earth surface detection^1^, wind speed measurements^2^, building construction^3^, mining^4^, forestry^5^, and robotics^6^. However, LiDAR technology gained tremendous popularity after the US Defense Advanced Research Projects Agency announced the grand challenge competition in 2004, which accelerated the development of completely autonomous vehicles that could navigate roads. This challenge emerged as a response to a congressional mandate that demanded a third of US military ground vehicles to be unmanned by 2015^7^. The competition exposed some flaws of the camera-based system and placed LiDAR sensors in the spotlight^8^. The camera-based systems have limitations in resolution and depth recognition capabilities^9,10^. Moreover, detecting 3D information without high-quality images is challenging^11,12^. Furthermore, the camera’s most obvious limitation is the lighting, which means that the camera cannot obtain reliable data from relatively or completely dark scenes or objects. Therefore, using LiDAR-based sensors in an obstacle avoidance system is necessary^8^. Currently, the rotating LiDAR sensors are the most mature imaging technique being used by the automotive industry^13–15^. The rotating scanning mechanism is popularly used by many commercial LiDAR sensors because it brings straight and parallel scan lines with a consistent scanning speed to generate a wide field of view (FoV). Thus, LiDAR systems have been used in autonomous vehicles for high-resolution simultaneous localization and mapping (SLAM) positioning and 3D model generation to provide a better sense the vehicles’ surrounding physical environment.

The rotating LiDAR system can provide a 360° horizontal FoV, achieved through a mechanical rotation system that spins the scanning part. According to the vertical scanning mechanism, LiDAR systems can be divided into two categories (i.e., multichannel and microelectromechanical systems [MEMS] mirror-based).

For the multichannel LiDAR system, the vertical FoV is defined by the number of existing emitter/receptor pairs. The single-line scanning only has one transceiver channel in the vertical direction. Thus, the scanning FoV is very limited. It is mainly used for anticollision in the forward direction of autonomous vehicles or for support images to generate the depth map^16^. Multichannel scanning LiDAR system refers to the multiple lasers that transit lights in the vertical direction to extend the vertical FoV by adding the FoV of these lasers. Compared with the single-line scanning LiDAR system, better vertical detection performance can be achieved. For example, the use of multichannel LiDAR system to detect pedestrians can often achieve higher accuracy than single-line LiDAR detection^17^. In 2007, Velodyne released the first 64-channel based on a rotation platform^18^, which has dominated the self-driving car market for a decade. This kind of LiDAR system allows for 360° horizontal detections and a fast scanning speed (one million samples per second at 15 Hz)^19^. However, their vertical angular resolution is limited by the amount of transmitter and receiver pairs at certain FoV. Thus, an improvement in the vertical angular resolution means the high cost of more transmitters and receivers. A multichannel LiDAR system in a self-driving car can cost up to USD 75,000^20^, making it the most expensive element in a self-driving car^9^. The current LiDAR solutions based on multichannel scanners are either with limited vertical resolution or costly.

Fortunately, MEMS technology provides a viable alternative. The MEMS mirrors have already gained enormous commercial success in projectors, displays, and fiber optic communications^21^. Being small, steer light in free space, and continuous switch light is the most critical characteristics of the MEMS mirror. In 2D MEMS mirror-based LiDAR systems, only the mirror plate of the MEMS device moves. Thus, MEMS-based LiDARs are often referred to as quasi-static state LiDAR. There are various technologies with MEMS mirrors that have been developed, including using 2D MEMS mirrors with multiple laser diodes^22^, multiple 1D resonant scanning MEMS mirrors^23^, and 2D MEMS mirror-based LiDAR systems (using only one laser diode)^24^. The scanning FoV of LiDARs is typically limited due to the constraint of MEMS mirror vibration angle. For the extension of horizontal FoV, some of the above research works use multiple laser sources or use multiple MEMS mirrors in LiDAR systems. A 2D quasi-static MEMS mirror with scanning frequencies in the horizontal and vertical directions of 5 Hz and 1.3 kHz, respectively, was applied in a LiDAR system^22^. In that work, a LiDAR system with 256 × 64 (horizontal × vertical) depth image resolution and 45° × 11° (horizontal × vertical) FoV was realized. In addition, the developed system used multiple laser diodes and associated lenses to cover a scanning angle of 45°. However, the system has a complex design due the control of several laser diodes with relevant driving circuits and also, adjustment of individual and mutual lenses’ position to ensure optimum focus on the MEMS mirror. To increase the horizontal FoV, another LiDAR system was developed using three 1D resonant scanning MEMS mirrors^23^. They achieved an angular resolution of 0.2° × 0.59° (horizontal × vertical) and a FoV of 60°×10° (horizontal × vertical), which benefited from the larger mechanical angle of the resonant scanning MEMS mirror in the horizontal direction. However, the use of the MEMS mirror at the receiver side limits the aperture (only 60π mm^2^) of the receiver system, which leads to lower backscattering energy and resulting in lower vertical edge quality of the point cloud and even lower vertical FoV. Moreover, closely similar characteristics of the two horizontal scanning MEMS mirrors are required to achieve synchronization. For the simpler structure LiDAR system reported in^24^, the 2D MEMS mirror enabled the system to achieve an angular resolution of 0.05° × 0.13° (horizontal × vertical) at a scanning rate of 100 Hz × 1 Hz (horizontal × vertical). However, the FoV of the system is only 5.78° × 6.36° (horizontal × vertical). The detailed specifications of the above systems are compared in Table 1. In previous works, authors have highlighted the fundamental problems of 2D MEMS mirror-based LiDAR systems. The FoV of the MEMS scanners remains as one of the most critical issues to be addressed.

**Table 1:** Comparing of the performance and cost of various LiDARs.

LiDAR design	Company	Channel	Number of MEMS mirror	Weight (kg)	FoV (H × V)	Angular resolution (H × V)	Points per second	Refresh rate	Cost
Puck	Velodyne	16	–	1	360° × 30°	0.1° × 2°	600,000	5–20 Hz for 360$$^\circ$$	$8,000
HDL-64E	Velodyne	64	–	12.7	360° × 27°	0.08° × 0.4°	2,200,000	5–20 Hz for 360$$^\circ$$	$75,000
VLS-128	Velodyne	128	–	5.3	360° × 40°	0.1° × 0.11$$^\circ$$	4,800,000	5–10 Hz for 360°	$53,000
Niclass et al.^22^	Research work	3	1	–	45° × 11°	0.17° × 0.17°	163,840	10 Hz	–
Xu et al.^23^	Research work	1	3	–	60° × 10°	0.2° × 0.59°	97,223	19 Hz	–
Lee et al.^24^	Research work	1	1	–	5.78° × 6.36°	0.05°× 0.13°	25,000	1 Hz	–
Our LiDAR system	Research work	1	1	2	360° × 8.6°	0.07° × 0.027°	6,300	0.004 Hz for 360°	$1700

The area of the 2D MEMS mirrors in LiDAR systems are generally larger than 28 mm^2^ in order to accommodate the laser diode beam^9^, such as the 32 mm^2^ mirror used in^22^ and the 66 mm^2^ mirror used in^24^; but the highest scanning angle of these mirrors is only 8°. The 2D MEMS mirrors reported in^25^ and ^26^ can reach maximum 90° and and has a mirror surface of 64 mm^2^. However, when the size of the MEMS mirrors increase, their frequency response decreases, resulting in a reduction of resolution in LiDAR systems. To improve the resolution, the design of ^27^ reduces the mirror area so that the frequency of the mirror reaches 0.4 kHz × 21.3 kHz (horizontal × vertical). However, the smaller mirrors result in lower detection distances for LiDAR systems^28^. On the other hand, 1D MEMS mirrors are more mature and usually have wider scanning angles, larger apertures, and higher resonant frequencies, making them a good choice for LiDAR systems^9^. In our system discussed here, to achieve 2D scanning, the 1D MEMS mirror is combined with a rotation platform. The rotation platform is a mature and easy to control device which is independent of the MEMS mirrors. Moreover, the rotation platform could enable 360° horizontal scanning of the LiDAR system. Thus, compared to LiDAR systems using 2D MEMS mirror designs which require more complex system architecture and optical components, the rotation platform can significantly reduce the overall system complexity while providing 360° FoV capability.

In this work, we focus on combining the advantages of rotating LiDAR system and MEMS mirror to achieve a large FoV and an overall higher angular resolution at a more reasonable system cost. The MEMS mirror is placed in a self-designed single transceiver module and integrated with a 360° rotation platform to realize panoramic scanning. Compared with the multichannel-based LiDAR system, the system described is not limited by the number of laser sources and receivers, making it possible to achieve 0.07$$^\circ \times$$ 0.027° (horizontal $$\times$$ vertical) angular resolution and an FoV of 360$$^\circ \times$$ 8.6° (horizontal $$\times$$ vertical). With this configuration, a 360° FoV LiDAR system can be realized with a reasonable price (US$1,700). Finally, we have also developed an data processing program to convert the scanned data into a 3D point cloud image, and the generated image proves the complete function of a MEMS mirror-based LiDAR system.

This paper describes the principle, prototyping, and testing of the MEMS mirror-based LIDAR system as follows. Section 2 introduces the design and principle. Section 3 presents the modeling and calibration. Section 4 presents the test results. Discussion and Conclusions are summarized in Sections 5 and 6, respectively.

### Design and principle

**Figure 1:**
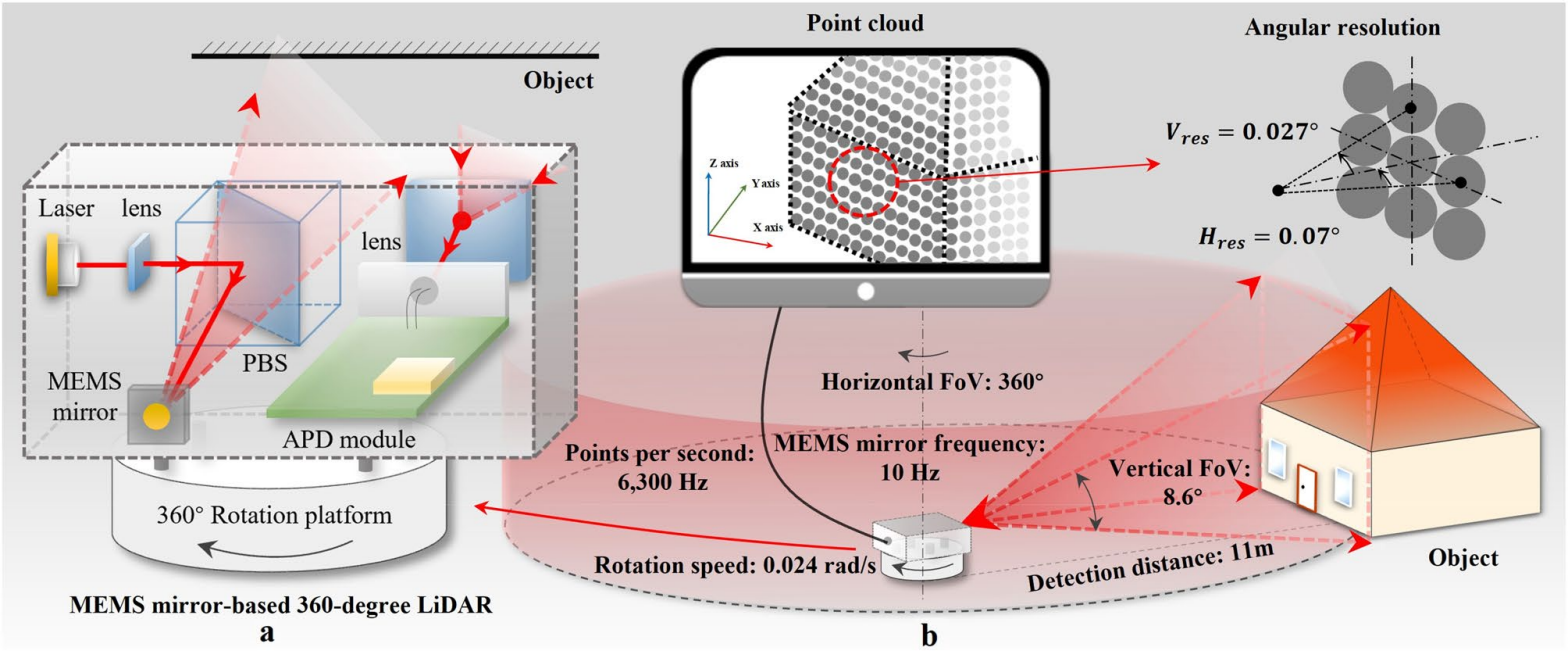
The MEMS mirror-based 360° LiDAR system. (**a**) The LiDAR system schematic. (**b**) The working scenario of LiDAR and specifications.

The basic structure of the pulsed time of flight (ToF) LiDAR system can be described as follows (Fig. 1a): a 905-nm laser (OSRAM SPLPL90) module with collimation generates a short laser pulse. This laser pulse is reflected by the single axis of the MEMS mirror to realize the vertical direction scanning, and the panoramic scanning is then achieved by the 360° rotation platform. The receiver will detect the reflected pulse from the target after the emitter process. Eventually, the laser pulse’s ToF, which is measured with the help of fast running counters, directly correlates with the distance between scenery and sensor, thus enabling three-dimensional environment perception.

The LiDAR system working scenario and specifications are shown in Fig. 1b. The angular resolution of a LiDAR system^29^ represents the smallest angular or linear separation between two points that can be resolved by the sensor. In other terms, it can be observed as the number of unit pulses per unit area. Thus, higher resolution sensors can create denser point clouds. The horizontal angular resolution of the current LiDAR system is expressed in the following equation.

1 $$\begin{array}{*{20}c} {H_{{{\text{res}}}} = \frac{\varphi }{{2 \times f_{{{\text{mirror}}}} }} } \\ \end{array}$$

where $H_{{{\text{res}}}}$ is the angular resolution of the horizontal direction, $\varphi$ is the rotation angle per second, and $f_{{{\text{mirror}}}}$ is the working frequency of the MEMS mirror. The horizontal angular resolution is mainly decided by the speed of the rotation platform and working frequency of the MEMS mirror (Eq. (1)), and the lower the ratio of $\varphi$ to $f_{{{\text{mirror}}}}$, the higher the angular resolution that can be obtained. Based on that, increasing the refresh rate of the system can be obtained by increasing the working frequency of the MEMS mirror and the speed of the rotation platform. The vertical angular resolution is shown in Eq. (2):

2 $$\begin{array}{*{20}c} {V_{{{\text{res}}}} = \frac{{V_{{{\text{FoV}}}} \times 2 \times f_{{{\text{mirror}}}} }}{N}} \\ \end{array}$$

where ${V}_{\mathrm{res}}$ is the angular resolution of the vertical direction, ${V}_{\mathrm{FoV}}$ is the vertical range FoV, and $N$ is the received points of the system. The number of received points is dependent on the frequency of the rate of the receiving system and the laser emission. The continuous motion angle of the MEMS mirror in the vertical direction allows for partial overlap of the emitted gaussian distribution of spots so that the system can obtain a denser point cloud.

#### MEMS mirror-based 360° LiDAR system

**Figure 2:**
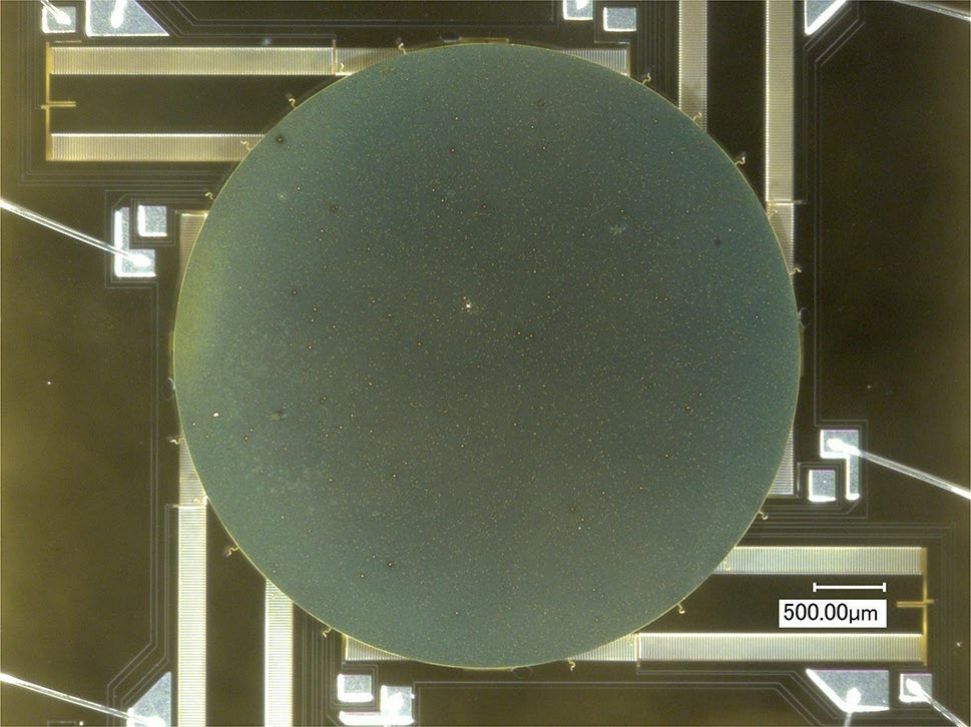
The structure of the MEMS mirror.

LiDAR system applications typically require an angular resolution of < 1 mrad, and the optical aperture of a MEMS mirror must be large for high-resolution scanning. However, the overall size of the scanner must be small for a compact^9^. The edge-emitting pulsed laser diodes will necessitate a scanning mirror plate with a minimum diameter of 3 mm^30^. Practical solutions can be achieved with reasonable trade-offs of performance and size with 4.6 mm diameter MEMS mirrors of the bonded design^31^ Due to the error caused by the production process, the parameters of each factory MEMS mirror may vary slightly, and actual measurements are required. Here, a MEMS mirror device (Mirrorcle S45868) with 5° mechanical angle is utilized and benefits from the point-to-point or quasistatic optical beam steering (Fig. 2). This means that any steady-state analog actuation voltage results in a specific steady-state analog angle of rotation of the mirror and consequently in a specific optical beam direction. Near DC (0 Hz), a one-to-one correspondence of actuation voltages and resulting angles were noted: it is highly repeatable with no measurable degradation over time. Also, the actuation range is amplified by adding transformers and lever structures^32^. In addition, it has excellent mechanical shock and vibration performance, less than a milliwatt power consumption, and is setup for standard silicon-based mass production.

#### Emitter and receiver module

**Figure 3:**
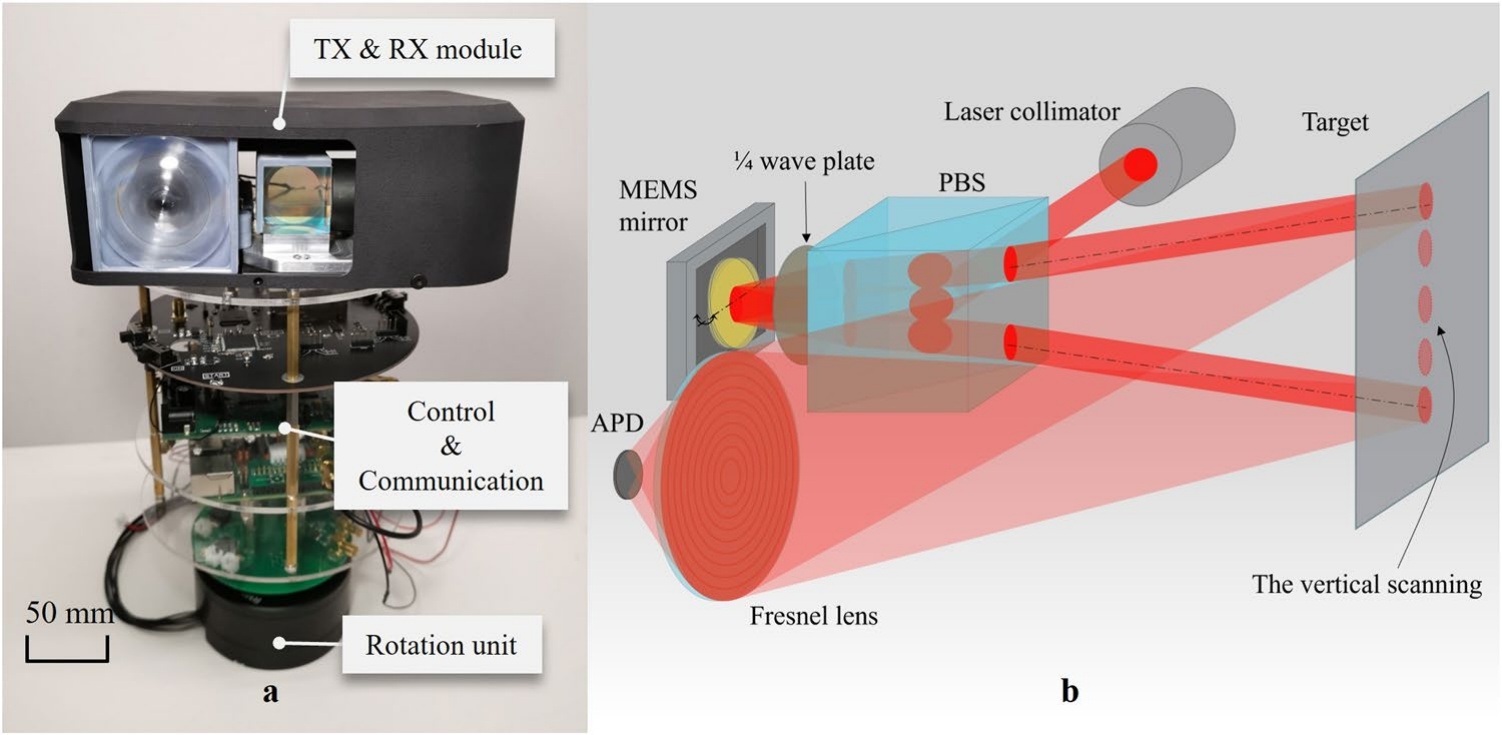
The LiDAR system layout. (**a**) The structure of 360° LiDAR system. (**b**) The schematic of TX and RX modules.

Figure 3a shows the overall structure of the developed LiDAR system prototype of the current study. The top layer is the emitter and receiver module, and the biaxial architecture was applied (Fig. 3b). This biaxial setup is more optimal for use with MEMS mirrors because the transmitter can be separately designed without receive-light considerations. However, 905 nm is within the responsivity of a silicon-based photodetector, and low-cost receivers can be used to build these systems. These components are relatively low cost compared to IR-Mid IR sources (e.g., 1550-nm wavelengths), which require expensive InGaAs receivers. Nevertheless, all those wavelengths are suitable for the diameter of around 5 mm and gold-coated mirror and have been successfully integrated into the system designs.

The receiving part of the LiDAR system is crucial to achieving the required signal-to-noise ratio, maximum distance, and FoV requirements. In the shown receiver signal chain, the receiver circuits’ main purpose is to amplify the received electrical current from the array of photodiodes with the help of high-performance amplifiers. This amplification circuit has to implement not only a high dynamic range (to detect targets in the near and far vicinities) but shall also support adjustable gain settings, short recovery times (to detect a weak pulse directly after a strong pulse), and low electrical/optical. Thus, the bias power supply is controlled with a thermosensor to keep the avalanche photodiode (APD) gain constant, and the ripple noise usually inherent to high-voltage power supplies is also minimized.

The transmitters are paired with the MEMS mirror in a physically offset location from the receiver. Compared with the coaxial LiDAR scan, the key benefit of this setup is that the receiver can be designed with the “arbitrarily large” optics because it is not constrained by the scanning element^9^. The relationship between the optics of the receiver and received signal power can be expressed as follows:

3 $$\begin{array}{*{20}c} {P_{r} = P_{s} \eta_{t} \times \frac{\rho }{{r^{2} }} \times \frac{{\pi D^{2} \eta_{r} }}{2} } \\ \end{array}$$

where $P_{r}$ and $P_{s}$ represent peak power of the returned and emitting pulse, $\eta_{t}$ and $\eta_{r}$ represent the efficiency of the transmitter optical and receiver optical, $\rho$ is the reflectivity of the target object, $r$ is range from the transmitter to the target, and $D$ is the receiver aperture diameter. From the equation, with the same receiving signal power to APD, the larger receiving optics’ apertures $D$ allow for longer distances $r$. The receiver aperture may be 25 mm or larger as needed while the scanning element is only 4.6 mm in diameter of the MEMS mirror due to the biaxial architecture. When using a single thin lens to collect returning light and placing the APD right on the focus of the lens, the half-angle FoV can be expressed as:

4 $$\begin{array}{*{20}c} {\tan \theta = \frac{d}{2f}} \\ \end{array}$$

where $\theta$ is the half-angle FoV, $f$ is the focal length of the lens, and $d$ is the APD diameter. Given the fixed APD size from the equation, a lens with smaller focal lengths is desired to increase the FoV angle. In this setup, a Fresnel lens with a 50-mm diameter and a 10-mm focal length is used to collect the back-scattered laser beam.

#### System control and communication

**Figure 4:**
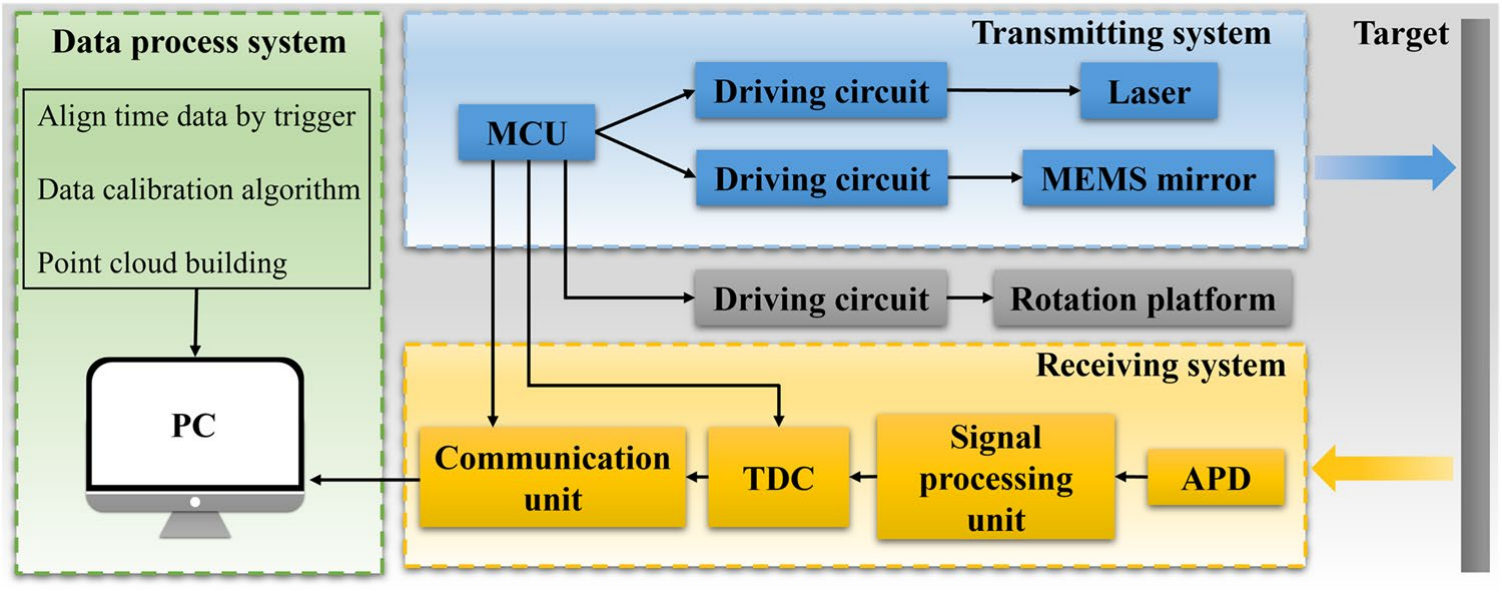
The LiDAR system schematic.

The schematic of the control and communication unit is shown in Fig. 4. The main function of the controlling unit is to generate control and trigger signals to drive the devices. An STM32 MCU serves as the system controller, whose functions include laser controlling, MEMS mirror controlling, rotation platform controlling, and trigger signal configuration. Each axis of the MEMS mirror actuated is by two quadrant devices^32^, the transient device performance and control schemes for optimizing device characteristics (e.g., settling time), which are schemes for closed-loop control of MEMS mirror using PID or adaptive controllers^33^ have been proposed, which require position sensors and often complex circuitry. Extremely high repeatable accuracy of laser beam steering in the open-loop method is inherent in the construction of the MEMS mirror itself. These MEMS mirrors are frequently employed in real-world imaging and detection applications with open-loop driving due to the high repeatability of the static and dynamic responses because of their pure single crystal-silicon structure and electrostatic driving. The open-loop control schemes using various filters and pulse-shaping are simpler to implement and competent for many applications. For example, Hah et al.^34^ reported a 120-μs settling time for small micromirrors (137 × 120 μm^2^).

The open-loop driving method of the MEMS mirror can be described as an approximate inverse system of the MEMS model. In the simplest form, the integrated MEMS device can be represented as a linear time-invariant second-order system^35^. The linearization is achieved by applying a voltage to all rotator segments and an additional voltage difference between opposing rotator segments to obtain a proportional rotator position. Therefore, the linear model assumes voltage ${V}_{\mathrm{in}}$ as the command input and mirror angle $\theta$ as the output can be described in the following equation:

5 $$\begin{array}{*{20}c} {H\left( s \right) = K \cdot \frac{{\omega_{n}^{2} }}{{s^{2} + 2 \cdot \zeta \cdot \omega_{n} \cdot s + \omega_{n}^{2} }}} \\ \end{array}$$

where $H\left( s \right){ }$ is mechanical spring-mass system response, $\omega_{n} { }$ is the undamped natural frequency of the system and $\zeta$ is the damping ratio, and the constant $K$ provides the conversion from momentary input voltage $V_{{{\text{in}}}}$ to mirror angle $\theta$. The $V_{{{\text{in}}}}$ is generated by the 12-bit digital-to-analog converter (DAC) channel of the controller system, and the analog output voltage of the DAC channel can be expressed in the following equation:

6 $$\begin{array}{*{20}c} {V_{{{\text{in}}}} = V_{{{\text{REF}}}} \times \frac{{{\text{DOR}}}}{4,095} } \\ \end{array}$$

where $V_{{{\text{REF}}}}$ is the input reference voltage. The DOR is the predefined value for linearly transformed $V_{{{\text{in}}}}$, and the analog voltage $V_{{{\text{in}}}}$ between 0 to $V_{{{\text{REF}}}}$ is determined. With the characteristics of quasistatic MEMS mirror, the momentary mirror angle can be determined by a certain $V_{{{\text{in}}}}$ value. Thus, the MEMS mirror angular motion can be obtained by continuous $V_{{{\text{in}}}} \left( t \right)$ and can be described in the following equation:

7 $$\begin{array}{*{20}c} {\theta = a_{1} \sin \left( {a_{2} \left( {t - t_{0} } \right) + a_{3} } \right) + a_{4} } \\ \end{array}$$

where $\theta$ is the momentary optical polar angle of MEMS mirror, ${t}_{0}$ is the absolute starting time, and $t$ is the absolute time for each point. The parameters ${a}_{1}$, ${a}_{2}$, ${a}_{3}$, and ${a}_{4}$ describe the MEMS mirror scan motion in the vertical direction. The trigger signal was generated by MCU with synchronization as soon as the mirror rotates at a specific position to indicate the start of the MEMS mirror’s scanning period. In addition, the MEMS mirror sinusoidal motion causes the density of the samples to be higher at the ends of the scanning range than in the middle region. Therefore, controlling the pulse emission of the laser to correspond to a specific interval of the mirror trajectory is necessary to utilize a more uniform mirror motion. It will avoid the above problem but will also reduce the FoV in the vertical direction.

The absolute time for each trigger pulse will be recorded by the communication unit. As the optical angle of the MEMS mirror changes according to Eq. (8), a few points would be sufficient to calculate the relationship between optical angle and time. After receiving the trigger pulse, the communication unit would start to receive the total time difference (TTD) from the time-to-digital converter (TDC) through the Serial Peripheral Interface protocol. Simultaneously, the rotation platform would start working in the 360° scanning mode. The TDC would calculate the TTD based on the input raw laser signal and processed APD signal, which contains the ToF and time delay inside the LiDAR system. After the scanning process, the unit would obtain the angular velocity from the rotation platform. Finally, the optical angle trigger, TTD, and the angular velocity information would be transmitted to the PC, which would calculate the position for each point and execute a calibration program to regulate the whole point cloud.

### Modeling and calibration

**Figure 5:**
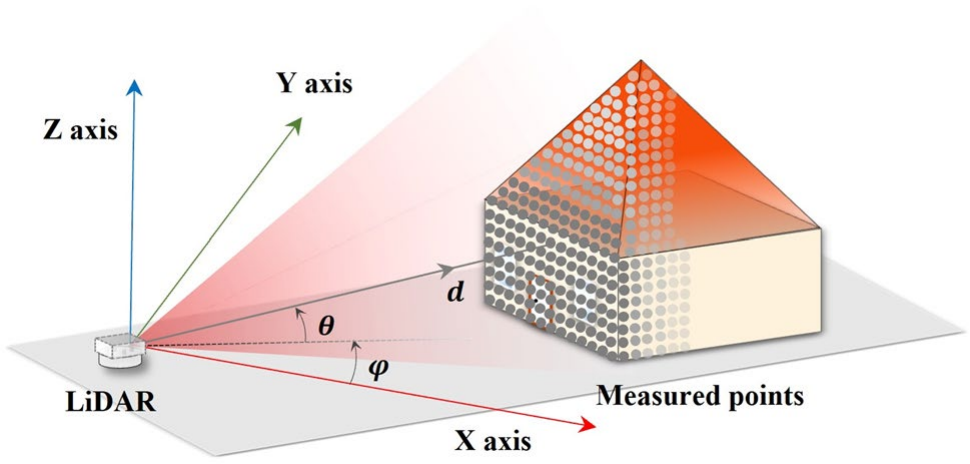
LiDAR system application scenario model.

Figure 5 illustrates how a point cloud position is calculated. The position of each point is determined by the distance from the origin and the two angles between the laser path and the coordinates, which is calculated as follows:

8 $$\begin{array}{*{20}c} {\left[ {\begin{array}{*{20}c} X \\ Y \\ {\begin{array}{*{20}c} Z \\ 1 \\ \end{array} } \\ \end{array} } \right] = \left[ {\begin{array}{*{20}c} {\begin{array}{*{20}c} {d \times \cos \left( \theta \right) \times \cos \left( \varphi \right)} \\ {d \times \cos \left( \theta \right) \times \sin \left( \varphi \right)} \\ {d \times \sin \left( \theta \right)} \\ \end{array} } \\ 1 \\ \end{array} } \right]} \\ \end{array}$$

where $d$ is the distance between the object point to the center of the LiDAR system; $\varphi$ is the azimuthal optical angle; $\theta$ is the polar optical angle; and *X*, *Y*, and *Z* are the position values of the point in Cartesian coordinate.

The angle information depends on the rotation platform and the MEMS mirror angular motion, the rotation platform motion with $\varphi$ can be defined as:

9 $$\begin{array}{*{20}c} {\varphi = \omega \times \left( {t - t_{0} } \right)} \\ \end{array}$$

Combining Eqs. (8) to (10) and (13), each point in the point cloud could be expressed using the 360° scanning mode as in the following equation.

10 $$\begin{array}{*{20}c} {\left[ {\begin{array}{*{20}c} X \\ Y \\ {\begin{array}{*{20}c} Z \\ 1 \\ \end{array} } \\ \end{array} } \right] = \left[ {\begin{array}{*{20}c} {\begin{array}{*{20}c} {\frac{{\left( {{\text{TTD}} - a_{6} } \right)}}{{a_{5} }} \times \cos \left( {a_{1} {\text{sin}}\left( {a_{2} \left( {t - t_{0} } \right) + a_{3} } \right) + a_{4} } \right) \times \cos \left( {{\upomega }\left( {t - t_{0} } \right)} \right)} \\ {\frac{{\left( {{\text{TTD}} - a_{6} } \right)}}{{a_{5} }} \times \cos \left( {a_{1} {\text{sin}}\left( {a_{2} \left( {t - t_{0} } \right) + a_{3} } \right) + a_{4} } \right) \times \sin \left( {{\upomega }\left( {t - t_{0} } \right)} \right)} \\ {\frac{{\left( {{\text{TTD}} - a_{6} } \right)}}{{a_{5} }} \times \sin \left( {a_{1} {\text{sin}}\left( {a_{2} \left( {t - t_{0} } \right) + a_{3} } \right) + a_{4} } \right)} \\ \end{array} } \\ 1 \\ \end{array} } \right]} \\ \end{array}$$

**Figure 6:**
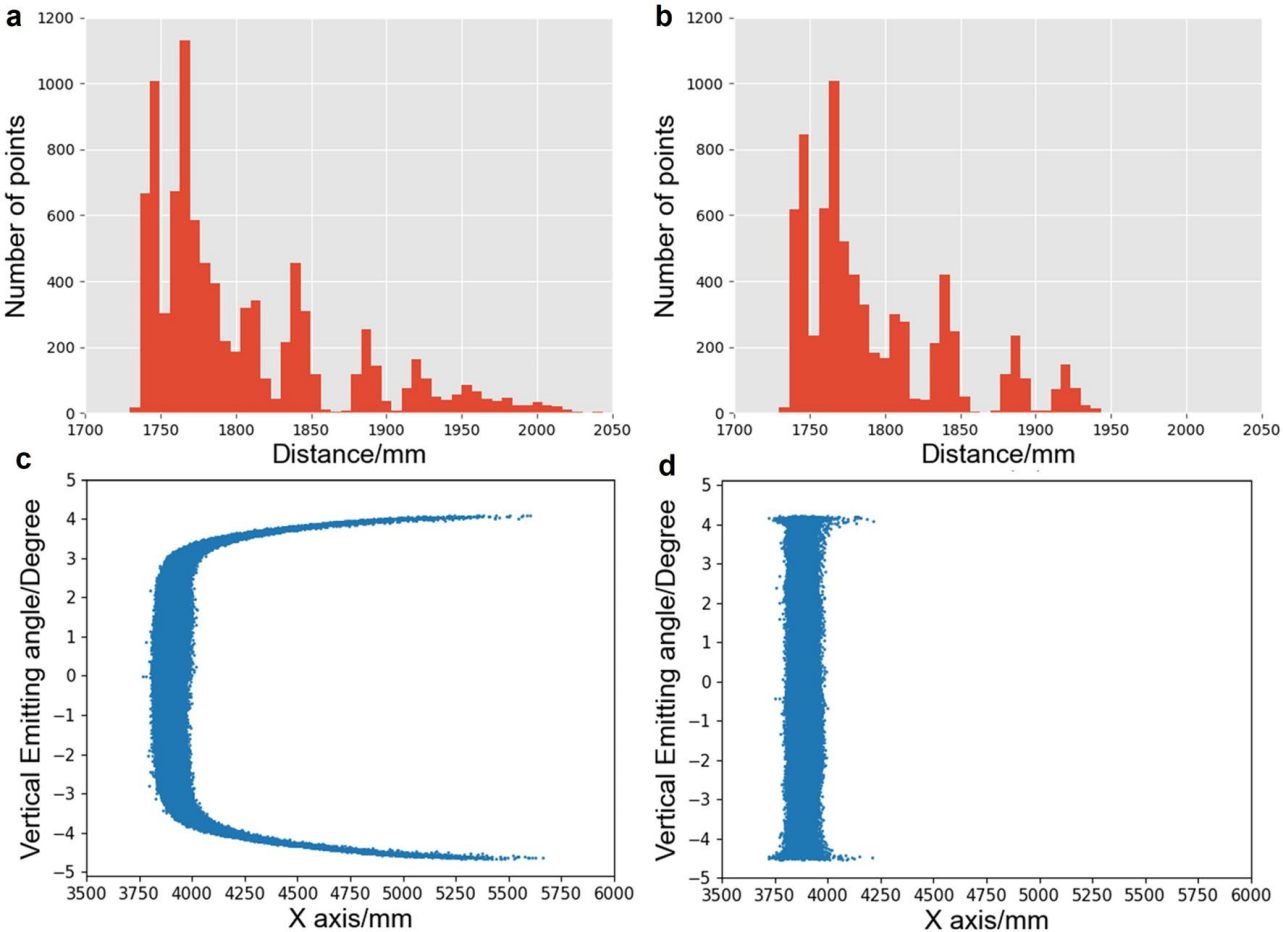
Scanned result calibration. (**a**) Original object histogram. (**b**) Histogram of the filtered point cloud. (**c**) Side view of the original point cloud. (**d**) Point cloud after the polynomial curve fitting.

When the LiDAR system scans the object’s edge, part of the laser spot may be on the object while the other is on the background object. This situation is called the veiling effect. To correct this distortion, the Scan Shadows filter was applied. The basic principle is that the perpendicular angle formed by the distorted and neighbor points is commonly larger than that formed by other normal points and their neighbor points. By analyzing the perpendicular angle of points, whether a point belongs to distorted points or not could be figured out. For each point, five neighbor points were found to calculate the perpendicular angle and decide whether to remove the point following the sum of five angles. The angle sum of point ${P}_{0}$ could be calculated as:

11 $$\begin{array}{*{20}c} {S_{0} = \mathop \sum \limits_{i = 1}^{5} \arccos \left( {\frac{{L^{{P_{0} P_{i} \;2}} + { }L^{{OP_{0} \;2}} - { }L^{{OP_{i} \;2}} }}{{2 \times L^{{P_{0} P_{i} }} \times L^{{OP_{0} }} }}} \right)} \\ \end{array}$$

where ${S}_{0}$ is the sum of perpendicular angles, $O$ is the origin point, ${P}_{0}$ is the inspection point, ${P}_{i}$ is the ${i}^{\mathrm{th}}$ neighbor point to inspection point, and $L$ is the distance between two points. Depending on the angle sum value for each point, points distorted by the veiling effect, the original depth map, and the filtered depth map of the object could be removed (Fig. 6a, b).

The internal building structure is much larger compared with the small object, and the whole scanning area would not exceed its edge. In that condition, the laser-receiving module performance in larger emitting angles influences the scanned result rather than the veiling effect. Some points would first be selected at the large-emitting angle area and then use to conduct the polynomial curve fitting. The error of the point cloud could be rectified after obtaining the relationship between the emitting angle and the distance on the *X*-axis. Moreover, Fig. 6c and d show the point cloud before and after the polynomial curve-fitting calibration.

### Experimental prototype implementation

**Figure 7:**
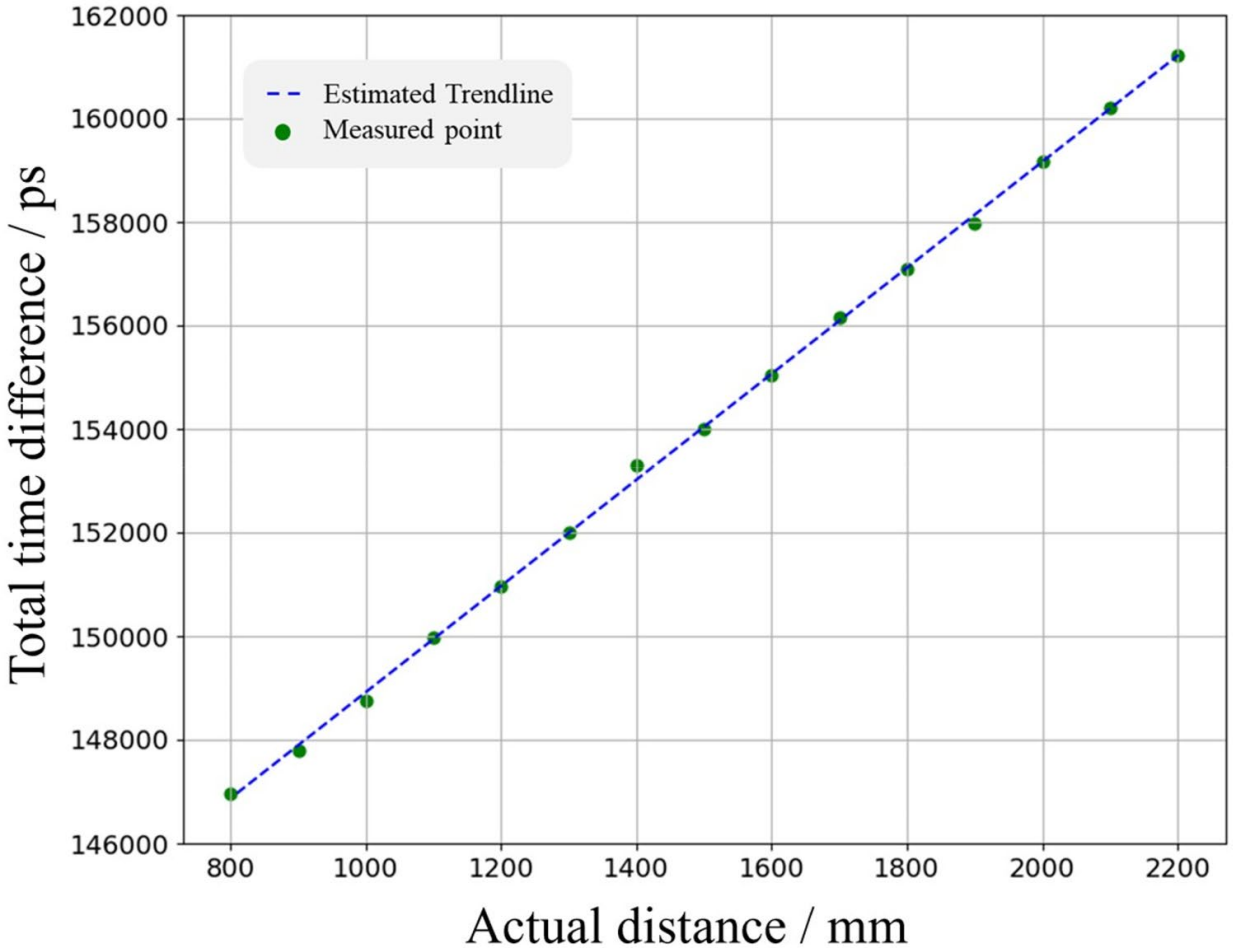
Measured TTD at each actual distance and the estimated trendline.

In the first experiment, the same static object was placed at different distances from the LiDAR in order to test the response of the system. A cardboard with a reflectivity around50% was used as the target object, and the calibration experiment was conducted with a static MEMS mirror. The relationship between the TTD and actual distance is shown in Fig. 7. The linear relationship between the TTD and actual distance is apparent. The calculated *R*^2^ of the fitted correlation is 0.99943. The correlation between the TTD and actual distance could be expressed as:

12 $$\begin{array}{*{20}c} {TTD = a_{5} \times d + a_{6} } \\ \end{array}$$

where the ${a}_{5}$ is the slope and ${a}_{6}$ is the intercept.

**Figure 8:**
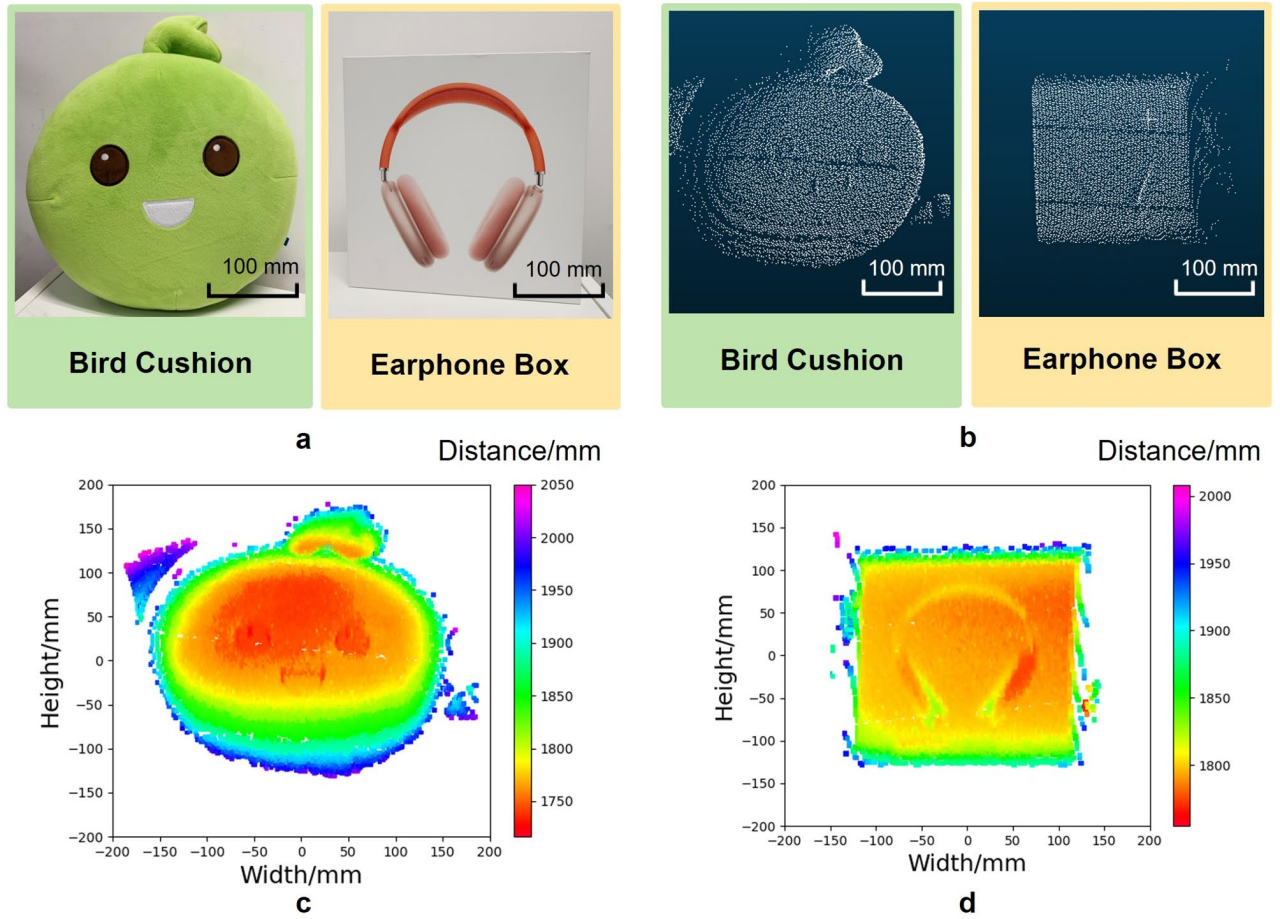
The scanning results. (**a**) The photograph of the scanned objects. (**b**) The scanned point-cloud. (**c**) The depth map of the bird cushion. (**d**) The depth map of the earphone box.

The measurements were conducted in an indoor room where two objects were placed around 360° LiDAR system (Fig. 8a). The main source of ambient light was fluorescent lamps. Thus, a filter was applied in front of the APD to allow only 905 nm wavelength light to pass through. Two multiplanar targets with different materials and geometric shapes were placed around the LiDAR system from 2 m. A front view of the point cloud obtained by 360° LiDAR system after the calibration is shown in Fig. 8b, and the surface formed by the two objects is revealed. The angular resolution reached 0.07°× 0.027°(horizontal $\times$ vertical). Moreover, Fig. 8c and d are the depth map extracted from the point cloud, in which different colors represent the distance from the point to the original center.

**Figure 9:**
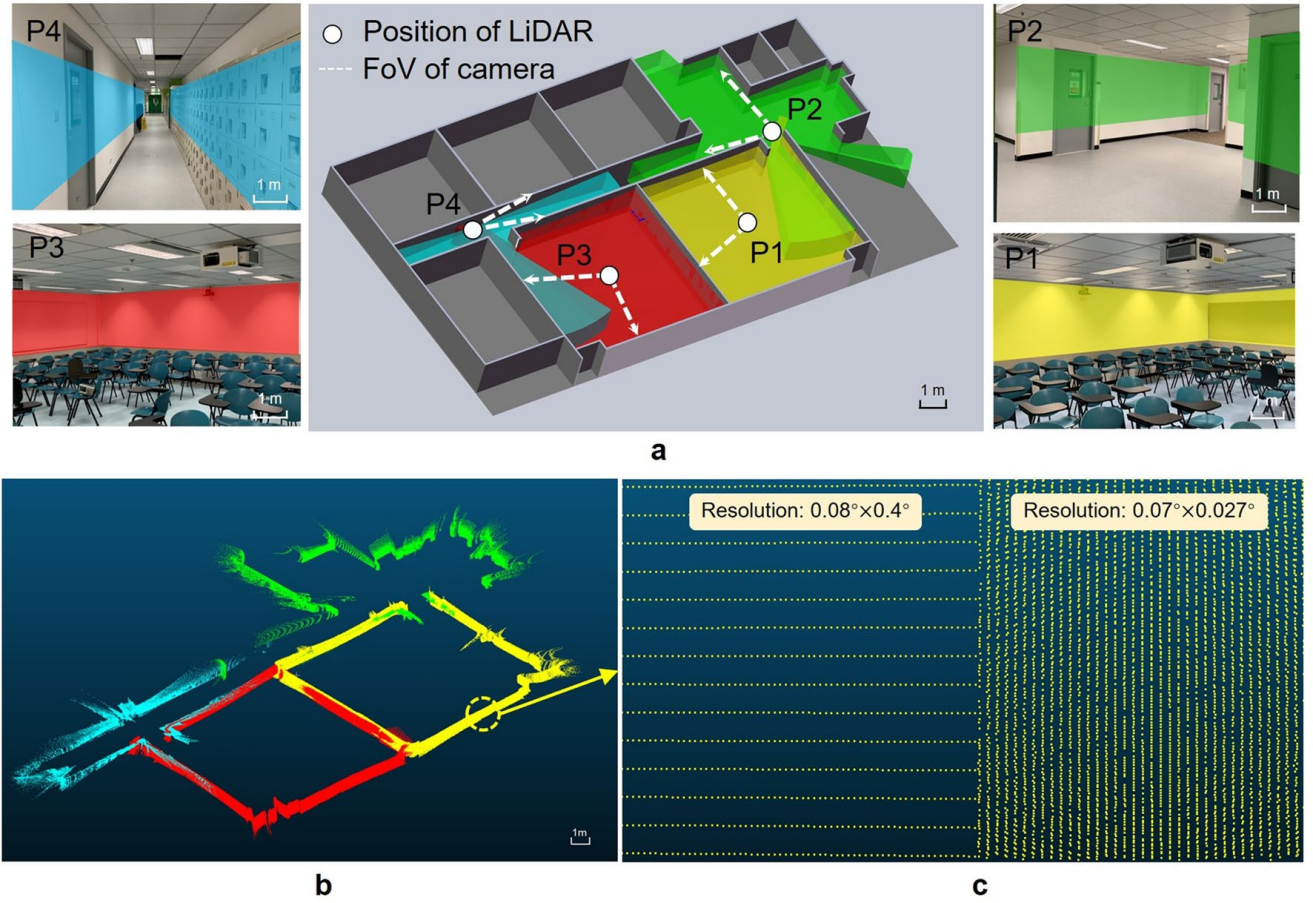
The space scanning results. (**a**) 3D structure of rooms and corridors. (**b**) The isometric view of the aligned point cloud. (**c**) The comparison of scanning results. *Left* simulation results of Velodyne HDL-64; *right* the LiDAR system of the current study.

The scanning experiment was conducted in a larger space to further demonstrate 360° scanning performance. The experimental procedure is elaborated as follows: firstly, the LiDAR system is located at P1 (Fig. 9a) after the 360° scanning process, moved to the next point P2 to conduct the same scanning process, and the same was conducted at P3 and P4. Different point clouds, which represent each scanned building structure, were then obtained using the algorithm to align these point clouds and merged into a point cloud map. In addition, Fig. 9b shows the isometric view of the merged point cloud. Four-point clouds show different colors. From the results, the shape of the rooms and corridors can be classified. Based on the above analysis and experiments, the feasibility of this MEMS mirror-based 360° LiDAR system design can be proved. The comparison of the current point cloud result with the simulated Velodyne HDL-64 point cloud result is shown in Fig. 9c. The results of the current study are denser with higher angular resolution and may show more details for the same wall area and distance.

## Discussion

**Figure 10:**
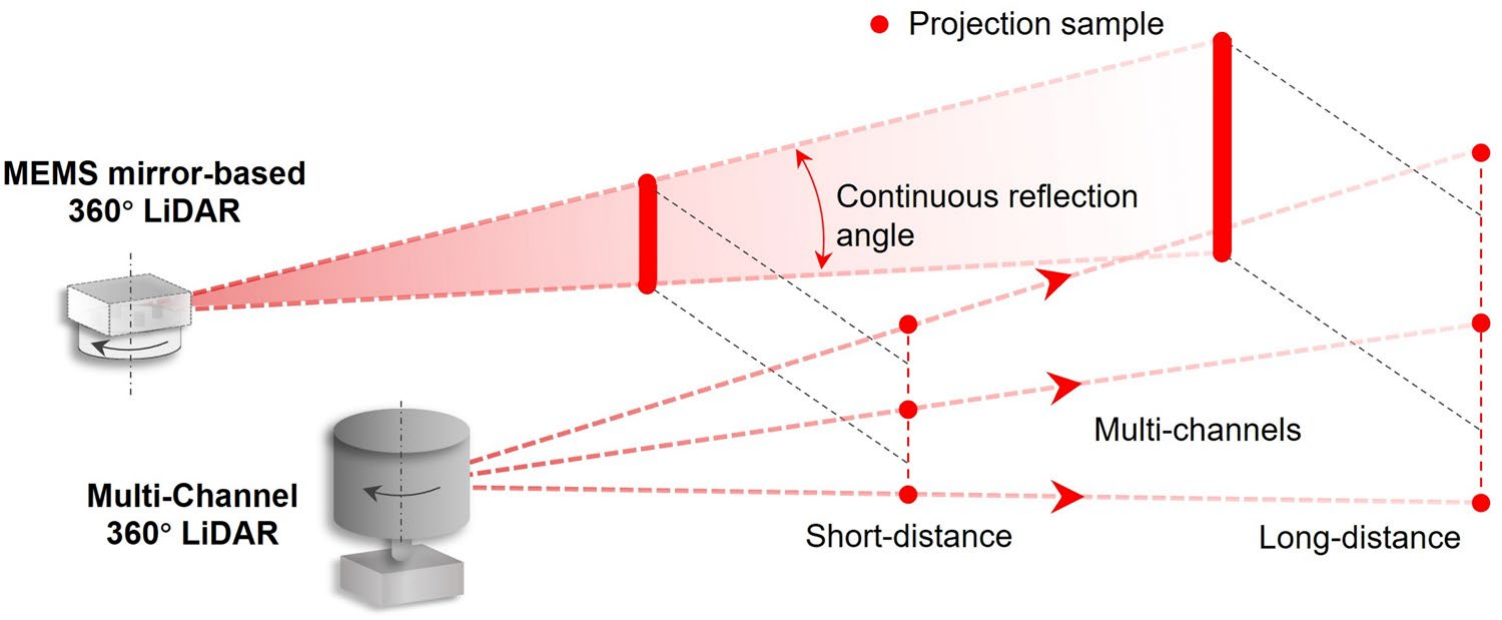
The comparison of the scanning mechanism between MEMS mirror-based and multichannel LiDAR system.

The vertical angular resolution can be significantly improved by increasing the working frequency of the laser with continuously changing the reflection angle of the MEMS mirror (Fig. 10). The amount of transceiver is limited by the size and cost. Therefore, the number of pulses per unit area of MEMS mirror-based LiDAR system is much higher than that of multichannel LiDAR system.

Table 1 compares the 3D LiDAR system prototype presented in this paper with three commercial 3D LiDAR system from Velodyne. Moreover, Table 1 shows that the LiDAR system prototype of the current study has a much higher angular resolution (i.e., 13.8 times better than Velodyne HDL-64E) and a much lower cost. In addition, its weight is less than the Velodyne LiDAR sensor with 64 and 128 channels. In future mass production, the price of the LiDAR system could probably be lower than 20% of Velodyne Puck, and its weight could be promisingly lightened to 1 kg after further integration. However, it showed a much lower point per second rate, but this limit can be addressed by improving the communication unit with an FPGA-based system^36^. Moreover, the vertical angular resolution of LiDAR system will also have a significant improvement by increasing the points per second rate according to Eq. (2).

With the technological development of commercial electronic equipment needed for LiDAR system, there will be a greater improvement in enhancing the performance of MEMS mirror-based LiDAR systems, such as refresh rate and FoV. The refresh rate and FoV of the 3D LiDAR system presented here is lower than those in the market. For the possibility of different application scenarios, the refresh rate and FoV need to be considered together. For example, the Puck (Velodyne) has a vertical FoV of 30° and an average refresh rate of 10 Hz for autonomous driving applications. According to Eqs. (1) and (2) by using a rotation platform with higher rotation rate, and faster and larger scanning angle MEMS mirror, it is theoretically possible for our design to achieve a higher system refresh rate and larger vertical scanning FoV. If a more advanced commercially available MEMS mirror (e.g., the OPUS Microsystems model 7200) with 52 kHz drive frequency and up to 40° fast axis FoV can be used in the future, the refresh rate of the LiDAR system potentially can be increased to 10 Hz with 0.07° horizontal angular resolution. Furthermore, a refresh rate of 20 Hz can be achieved by applying faster MEMS mirrors that have been developed^37^. To keep the 0.027° of vertical angular resolution at a 30° vertical FoV and 10 Hz refresh rate the points per second of the system need to reach 57.1 MSPS, which means that the post-stage circuit of the system also need to be redesigned.

The FoV of MEMS mirror-based LiDAR systems depends on the angle of the MEMS mirror and the FoV of the receiver module. To increase in the vertical FoV of the current system, commercial MEMS mirrors with optical scan angles up to 60° (e.g., ZHIWEI C1130) can be implemented in the future. Therefore, the FoV of the proposed LiDAR system is mainly limited by the receiver module. An effective way to enhance the FoV of the receiver module is to use an APD with a larger effective area; however, this will also lead to lower bandwidth and reduce the accuracy of the LiDAR system. For this work, we selected an APD with an effective area of 1.5 mm, and used a Fresnel lens with a diameter of 50 mm and a focal length of 10 mm to achieve a vertical FoV of 8.6°. And, by controlling the MEMS mirror, a continuous motion angle of 10.2° in the vertical direction is realized to cover the FoV of the receiving module.

## Conclusion

In this study, a MEMS mirror-based 360° LiDAR system with high vertical scanning resolution has been demonstrated. For this LiDAR system, the horizontal scanning is achieved by a 360° rotation platform, while the vertical scanning is realized by a scanning MEMS mirror. A complete LiDAR system was realized by designing and implementing the optical path for the laser emitter and receiver, based on the horizontal and vertical scanning requirements, and developing the control and communication circuitry. According to the experimental result, the system achieved good performance in FoV (360° in horizontal direction and 8.6° in vertical direction) and angular resolution (0.07° in horizontal direction and 0.027° in vertical direction). Compared with the commercial Velodyne HDL-64 LiDAR sensor, the vertical angular resolution of the our system is improved by 13.8 times. This feature could enable the further realization of high-quality panoramic scanning and an affordable solution for autonomous driving, robotics navigation, indoor surveying, etc.

## Data Availability

The datasets generated during and/or analyzed as part of the current study is available from the corresponding author on reasonable request.
